# Supplementary material for: Revisiting the role of the spindle assembly checkpoint in the formation of gross chromosomal rearrangements in Saccharomyces cerevisiae
Source: Genetics. 2024 Sep 12;228(3):iyae150. doi: 10.1093/genetics/iyae150 (PMC11538403; doi:10.1093/genetics/iyae150)

Table S1. Yeast strains used in this study.

Strain name	Genotype	Source
ZYY114	*MAT***a** *ura3Δ0 leu2Δ0 met15Δ0 lyp1Δ hxt13ΔURA3 mfa1::*P_{MFA1}*-HIS3 prb1ΔhphMX-50bp_ITS his3ΔkanMX*	This study
ZYY139	*MAT***a** *his3Δ1 ura3Δ0 leu2Δ0 met15Δ0 lyp1Δ hxt13ΔURA3 mfa1::*P_{MFA1}*-HIS3 prb1ΔhphMX*	This study
ZYY141	*MAT***a** *his3Δ1 ura3Δ0 leu2Δ0 met15Δ0 lyp1Δ hxt13ΔURA3 mfa1::*P_{MFA1}*-HIS3 prb1ΔhphMX-50bp_ITS*	This study
YYY28	*MAT***a** *his3Δ1 ura3Δ0 leu2Δ0 met15Δ0 lyp1Δ hxt13ΔURA3 mfa1::*P_{MFA1}*-HIS3 prb1ΔhphMX-50bp_ITS mad1ΔkanMX*	This study
YYY18	*MAT***a** *his3Δ1 ura3Δ0 leu2Δ0 met15Δ0 lyp1Δ hxt13ΔURA3 mfa1::*P_{MFA1}*-HIS3 prb1ΔhphMX-50bp_ITS mad2ΔkanMX*	This study
YYY20	*MAT***a** *his3Δ1 ura3Δ0 leu2Δ0 met15Δ0 lyp1Δ hxt13ΔURA3 mfa1::*P_{MFA1}*-HIS3 prb1ΔhphMX-50bp_ITS mad3ΔkanMX*	This study
YYY22	*MAT***a** *his3Δ1 ura3Δ0 leu2Δ0 met15Δ0 lyp1Δ hxt13ΔURA3 mfa1::*P_{MFA1}*-HIS3 prb1ΔhphMX-50bp_ITS bub1ΔkanMX*	This study
YYY26	*MAT***a** *his3Δ1 ura3Δ0 leu2Δ0 met15Δ0 lyp1Δ hxt13ΔURA3 mfa1::*P_{MFA1}*-HIS3 prb1ΔhphMX-50bp_ITS bub3ΔkanMX*	This study
YYY24	*MAT***a** *his3Δ1 ura3Δ0 leu2Δ0 met15Δ0 lyp1Δ hxt13ΔURA3 mfa1::*P_{MFA1}*-HIS3 prb1ΔhphMX-50bp_ITS bub2ΔkanMX*	This study
YYY30	*MAT***a** *his3Δ1 ura3Δ0 leu2Δ0 met15Δ0 lyp1Δ hxt13ΔURA3 mfa1::*P_{MFA1}*-HIS3 prb1ΔhphMX-50bp_ITS bfa1ΔkanMX*	This study
YYY39	*MAT***a** *his3Δ1 ura3Δ0 leu2Δ0 met15Δ0 lyp1Δ hxt13ΔURA3 mfa1::*P_{MFA1}*-HIS3 prb1ΔhphMX-50bp_ITS kip1ΔkanMX*	This study
YYY37	*MAT***a** *his3Δ1 ura3Δ0 leu2Δ0 met15Δ0 lyp1Δ hxt13ΔURA3 mfa1::*P_{MFA1}*-HIS3 prb1ΔhphMX-50bp_ITS cik1ΔkanMX*	This study
YYY35	*MAT***a** *his3Δ1 ura3Δ0 leu2Δ0 met15Δ0 lyp1Δ hxt13ΔURA3 mfa1::*P_{MFA1}*-HIS3 prb1ΔhphMX-50bp_ITS bik1ΔkanMX*	This study
YYY43	*MAT***a** *his3Δ1 ura3Δ0 leu2Δ0 met15Δ0 lyp1Δ hxt13ΔURA3 mfa1::*P_{MFA1}*-HIS3 prb1ΔhphMX-50bp_ITS dyn1ΔkanMX*	This study
YYY90	*MAT***a** *his3Δ1 ura3Δ0 leu2Δ0 met15Δ0 lyp1Δ hxt13ΔURA3 mfa1::*P_{MFA1}*-HIS3 prb1ΔhphMX-50bp_ITS dyn3ΔkanMX*	This study
YYY45	*MAT***a** *his3Δ1 ura3Δ0 leu2Δ0 met15Δ0 lyp1Δ hxt13ΔURA3 mfa1::*P_{MFA1}*-HIS3 prb1ΔhphMX-50bp_ITS pac11ΔkanMX*	This study
YYY92	*MAT***a** *his3Δ1 ura3Δ0 leu2Δ0 met15Δ0 lyp1Δ hxt13ΔURA3 mfa1::*P_{MFA1}*-HIS3 prb1ΔhphMX-50bp_ITS jnm1ΔkanMX*	This study
YYY94	*MAT***a** *his3Δ1 ura3Δ0 leu2Δ0 met15Δ0 lyp1Δ hxt13ΔURA3 mfa1::*P_{MFA1}*-HIS3 prb1ΔhphMX-50bp_ITS nip100ΔkanMX*	This study
YYY41	*MAT***a** *his3Δ1 ura3Δ0 leu2Δ0 met15Δ0 lyp1Δ hxt13ΔURA3 mfa1::*P_{MFA1}*-HIS3 prb1ΔhphMX-50bp_ITS pac1ΔkanMX*	This study
YYY98	*MAT***a** *his3Δ1 ura3Δ0 leu2Δ0 met15Δ0 lyp1Δ hxt13ΔURA3 mfa1::*P_{MFA1}*-HIS3 prb1ΔhphMX-50bp_ITS kre28ΔkanMX*	This study
YYY96	*MAT***a** *his3Δ1 ura3Δ0 leu2Δ0 met15Δ0 lyp1Δ hxt13ΔURA3 mfa1::*P_{MFA1}*-HIS3 prb1ΔhphMX-50bp_ITS sgo1ΔkanMX*	This study
YYY47	*MAT***a** *his3Δ1 ura3Δ0 leu2Δ0 met15Δ0 lyp1Δ hxt13ΔURA3 mfa1::*P_{MFA1}*-HIS3 prb1ΔhphMX-50bp_ITS gim3ΔkanMX*	This study
YYY49	*MAT***a** *his3Δ1 ura3Δ0 leu2Δ0 met15Δ0 lyp1Δ hxt13ΔURA3 mfa1::*P_{MFA1}*-HIS3 prb1ΔhphMX-50bp_ITS gim4ΔkanMX*	This study
YYY71	*MAT***a** *his3Δ1 ura3Δ0 leu2Δ0 met15Δ0 lyp1Δ hxt13ΔURA3 mfa1::*P_{MFA1}*-HIS3 prb1ΔhphMX-50bp_ITS gim5ΔkanMX*	This study
YYY73	*MAT***a** *his3Δ1 ura3Δ0 leu2Δ0 met15Δ0 lyp1Δ hxt13ΔURA3 mfa1::*P_{MFA1}*-HIS3 prb1ΔhphMX-50bp_ITS pac10ΔkanMX*	This study
YYY110	*MAT***a** *his3Δ1 ura3Δ0 leu2Δ0 met15Δ0 lyp1Δ hxt13ΔURA3 mfa1::*P_{MFA1}*-HIS3 prb1ΔhphMX-50bp_ITS pfd1ΔkanMX*	This study
YYY75	*MAT***a** *his3Δ1 ura3Δ0 leu2Δ0 met15Δ0 lyp1Δ hxt13ΔURA3 mfa1::*P_{MFA1}*-HIS3 prb1ΔhphMX-50bp_ITS yke2ΔkanMX*	This study

YYY33	*MAT***a** *his3Δ1 ura3Δ0 leu2Δ0 met15Δ0 lyp1Δ hxt13ΔURA3 mfa1::*P_{MFA1}*-HIS3 prb1ΔhphMX-50bp_ITS ctf8ΔkanMX*	This study
YYY79	*MAT***a** *his3Δ1 ura3Δ0 leu2Δ0 met15Δ0 lyp1Δ hxt13ΔURA3 mfa1::*P_{MFA1}*-HIS3 prb1ΔhphMX-50bp_ITS dcc1ΔkanMX*	This study
ZYY162	*MAT***a** *his3Δ1 ura3Δ0 leu2Δ0 met15Δ0 lyp1Δ hxt13ΔURA3 mfa1::*P_{MFA1}*-HIS3 ho::CIN8-natMX prb1ΔhphMX*	This study
ZYY164	*MAT***a** *his3Δ1 ura3Δ0 leu2Δ0 met15Δ0 lyp1Δ hxt13ΔURA3 mfa1::*P_{MFA1}*-HIS3 ho::CIN8-natMX prb1ΔhphMX-50bp_ITS*	This study
ZYY210	*MAT***a** *his3Δ1 ura3Δ0 leu2Δ0 met15Δ0 lyp1Δ hxt13ΔURA3 mfa1::*P_{MFA1}*-HIS3 ho::CIN8-natMX prb1ΔhphMX mad1ΔkanMX*	This study
ZYY190	*MAT***a** *his3Δ1 ura3Δ0 leu2Δ0 met15Δ0 lyp1Δ hxt13ΔURA3 mfa1::*P_{MFA1}*-HIS3 ho::CIN8-natMX prb1ΔhphMX-50bp_ITS mad1ΔkanMX*	This study
ZYY198	*MAT***a** *his3Δ1 ura3Δ0 leu2Δ0 met15Δ0 lyp1Δ hxt13ΔURA3 mfa1::*P_{MFA1}*-HIS3 ho::CIN8-natMX prb1ΔhphMX mad2ΔkanMX*	This study
ZYY180	*MAT***a** *his3Δ1 ura3Δ0 leu2Δ0 met15Δ0 lyp1Δ hxt13ΔURA3 mfa1::*P_{MFA1}*-HIS3 ho::CIN8-natMX prb1ΔhphMX-50bp_ITS mad2ΔkanMX*	This study
ZYY216	*MAT***a** *his3Δ1 ura3Δ0 leu2Δ0 met15Δ0 lyp1Δ hxt13ΔURA3 mfa1::*P_{MFA1}*-HIS3 ho::CIN8-natMX prb1ΔhphMX mad3ΔkanMX*	This study
ZYY182	*MAT***a** *his3Δ1 ura3Δ0 leu2Δ0 met15Δ0 lyp1Δ hxt13ΔURA3 mfa1::*P_{MFA1}*-HIS3 ho::CIN8-natMX prb1ΔhphMX-50bp_ITS mad3ΔkanMX*	This study
YYY138	*MAT***a** *his3Δ1 ura3Δ0 leu2Δ0 met15Δ0 lyp1Δ hxt13ΔURA3 mfa1::*P_{MFA1}*-HIS3 ho::CIN8-natMX prb1ΔhphMX bub1ΔkanMX*	This study
YYY128	*MAT***a** *his3Δ1 ura3Δ0 leu2Δ0 met15Δ0 lyp1Δ hxt13ΔURA3 mfa1::*P_{MFA1}*-HIS3 ho::CIN8-natMX prb1ΔhphMX-50bp_ITS bub1ΔkanMX*	This study
YYY142	*MAT***a** *his3Δ1 ura3Δ0 leu2Δ0 met15Δ0 lyp1Δ hxt13ΔURA3 mfa1::*P_{MFA1}*-HIS3 ho::CIN8-natMX prb1ΔhphMX bub3ΔkanMX*	This study
YYY132	*MAT***a** *his3Δ1 ura3Δ0 leu2Δ0 met15Δ0 lyp1Δ hxt13ΔURA3 mfa1::*P_{MFA1}*-HIS3 ho::CIN8-natMX prb1ΔhphMX-50bp_ITS bub3ΔkanMX*	This study
YYY140	*MAT***a** *his3Δ1 ura3Δ0 leu2Δ0 met15Δ0 lyp1Δ hxt13ΔURA3 mfa1::*P_{MFA1}*-HIS3 ho::CIN8-natMX prb1ΔhphMX bub2ΔkanMX*	This study
YYY130	*MAT***a** *his3Δ1 ura3Δ0 leu2Δ0 met15Δ0 lyp1Δ hxt13ΔURA3 mfa1::*P_{MFA1}*-HIS3 ho::CIN8-natMX prb1ΔhphMX-50bp_ITS bub2ΔkanMX*	This study
ZYY214	*MAT***a** *his3Δ1 ura3Δ0 leu2Δ0 met15Δ0 lyp1Δ hxt13ΔURA3 mfa1::*P_{MFA1}*-HIS3 ho::CIN8-natMX prb1ΔhphMX bfa1ΔkanMX*	This study
ZYY192	*MAT***a** *his3Δ1 ura3Δ0 leu2Δ0 met15Δ0 lyp1Δ hxt13ΔURA3 mfa1::*P_{MFA1}*-HIS3 ho::CIN8-natMX prb1ΔhphMX-50bp_ITS bfa1ΔkanMX*	This study
ZYY206	*MAT***a** *his3Δ1 ura3Δ0 leu2Δ0 met15Δ0 lyp1Δ hxt13ΔURA3 mfa1::*P_{MFA1}*-HIS3 ho::CIN8-natMX prb1ΔhphMX ctf8ΔkanMX*	This study
YYY134	*MAT***a** *his3Δ1 ura3Δ0 leu2Δ0 met15Δ0 lyp1Δ hxt13ΔURA3 mfa1::*P_{MFA1}*-HIS3 ho::CIN8-natMX prb1ΔhphMX-50bp_ITS ctf8ΔkanMX*	This study
ZYY208	*MAT***a** *his3Δ1 ura3Δ0 leu2Δ0 met15Δ0 lyp1Δ hxt13ΔURA3 mfa1::*P_{MFA1}*-HIS3 ho::CIN8-natMX prb1ΔhphMX dcc1ΔkanMX*	This study
YYY136	*MAT***a** *his3Δ1 ura3Δ0 leu2Δ0 met15Δ0 lyp1Δ hxt13ΔURA3 mfa1::*P_{MFA1}*-HIS3 ho::CIN8-natMX prb1ΔhphMX-50bp_ITS dcc1ΔkanMX*	This study

Figure S1. Fold change in canavanine/5-FOA-resistance rate of the indicated strains. Same data as shown in Figure 1e, but plotted relative to the wild-type strain without an ITS. Error bars represent SEM (n = 3–6).

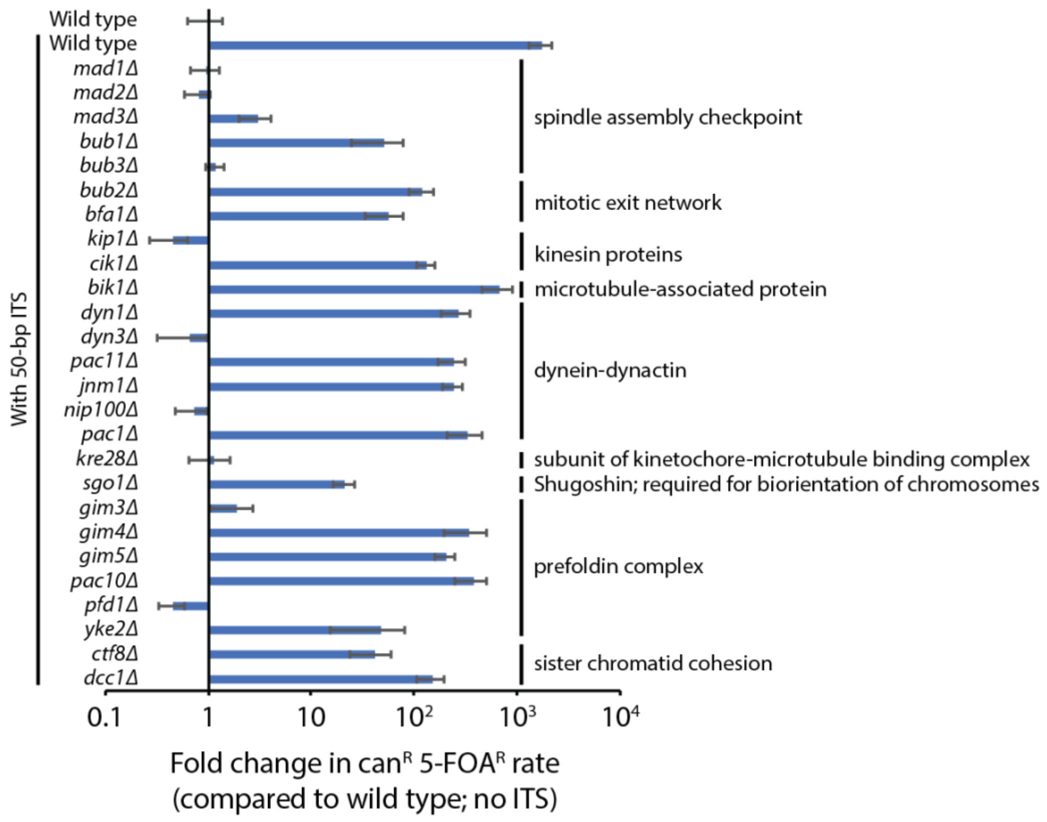

Supplement: iyae150_Supplementary_Data [file iyae150_supplementary_data.pdf]
